# Altered expression of glycobiology-related genes in Parkinson’s disease brain

**DOI:** 10.3389/fnmol.2022.1078854

**Published:** 2022-11-24

**Authors:** Jay S. Schneider, Garima Singh

**Affiliations:** Department of Pathology, Anatomy and Cell Biology, Thomas Jefferson University, Philadelphia, PA, United States

**Keywords:** Parkinson’s disease, glycolipid, sphingolipid, substantia nigra, putamen, gene expression

## Abstract

The precise mechanisms initiating and perpetuating the cellular degeneration in Parkinson’s disease (PD) remain unclear. There is decreased expression of the main brain gangliosides, and GM1 ganglioside in particular, in the PD brain along with decreased expression of the genes coding for the glycosyltranferase and the sialyltransferase responsible for the synthesis of these brain gangliosides. However, potentially important pathogenic mechanisms contributing to the neurodegeneration in PD may also include altered levels of expression of genes involved in glycosylation, sialylation and sphingolipid synthesis and metabolism. Although various studies have described pathological lipid and glycolipid changes in PD brain, there have been limited studies of expression of glycobiology-related genes in PD brain. The current study was performed as an initial attempt to gain new information regarding potential changes in glycoprotein and glycolipid-related genes in PD by investigating the gene expression status for select glycosyltransferases, sialyltransferases, sialidases, sphingosine kinases, and lysosomal enzymes in the substantia nigra and putamen from patients with PD and neurologically normal controls. Results showed altered expression of glycosyltransferase genes (*B3GALT2* and *B4GALT*1) potentially involved in microglial activation and neuroinflammation, sphingosine-1-phosphate (S1P) modulators (*SPHK1*, *SPHK2*, and *SGPL1*) involved in sphingolipid synthesis and metabolism, polysialyltransferase genes (*ST8SIA2* and *ST8SIA4*) that encode enzymes responsible for polysialic acid (polySia) biosynthesis, and the sialidase *NEU4*, expression of which has been linked to the clearance of storage materials from lysosomes. The data presented here underscore the complexity of the glycolipid/sphingolipid dysregulation in the PD brain and continued and expanded study of these processes may not only provide a greater understanding of the complex roles of aberrant glycosylation sialylation, and sphingolipid synthesis/metabolism in the pathophysiology of PD but may identify potential druggable targets for PD therapeutics.

## Introduction

Parkinson’s disease (PD) is a complex progressive neurodegenerative disorder primarily characterized by the loss of nigrostriatal dopamine-producing neurons in the substantia nigra. Although the majority of cases of PD are idiopathic in origin, several mechanisms have been proposed to explain the onset and progression of the neurodegeneration in PD including mitochondrial dysfunction, increased oxidative stress and oxidative damage, α-synuclein aggregation and associated toxicity, and lysosomal and autophagic dysfunction, among other potential contributing factors (Doria et al., 2016). In addition, various abnormalities in the content and composition of various lipids, including gangliosides, have been reported in PD brain [ex. (den Jager, 1969; Riekkinen et al., 1975; Seyfried et al., 2018)]. Recently, dysregulation of ceramide synthesis and metabolism have been suggested to play a role in PD-associated neurodegeneration (Plotegher et al., 2019) and lipdomic studies have reported PD-specific lipid alterations detected in brain and in plasma that have been suggested to promote PD-associated neurodegeneration at least in part through promoting α-synuclein aggregation, neuroinflammation, and dysfunction of autophagic processing [see Alecu and Bennett (2019) for review].

In addition to changes in lipid content and metabolism in the PD brain, some studies have also reported altered glycosylation and sialylation in PD brain (Videira and Castro-Caldas, 2018; Wilkinson et al., 2021). Altered levels of sialylation and fucosylation have also been reported in serum samples from PD patients and interestingly, mainly in male patients (Varadi et al., 2019). Additionally, many proteins are synthesized in the endoplasmic reticulum (ER) where many of them undergo glycoslylation and functionalization. ER stress has been suggested to be one of the pathological mechanisms contributing to PD (Mercado et al., 2013; Tsujii et al., 2015), and aberrant glycosylation has been suggested to contribute to an overload of the ER in PD brain with underglycosylated proteins (Videira and Castro-Caldas, 2018). Oxidative stress and inflammation have also been suggested to potentially trigger abnormal glycosylation in PD (Videira and Castro-Caldas, 2018).

Although various studies have described pathological lipid and glycolipid changes in PD brain, there have been limited studies of expression of glycobiology-related genes in PD brain (see (Alecu and Bennett, 2019) for review) and how dysregulation of the expression of these genes may contribute to PD-like neurodegeneration. In contrast, genes related to glycobiology have been examined in Huntington’s disease (HD) transgenic mice as well as in the caudate nucleus from human HD subjects (Desplats et al., 2007) where a number of glycosyltransferases and sialyltransferases were found to be significantly changed compared to normal controls. In particular, ganglioside metabolism genes *ST3GAL5*, *ST8SIA3*, *B4GALNT1*, and *ST3GAL2* had significantly decreased expression in HD caudate compared to control caudate (Desplats et al., 2007). We previously described significantly decreased expression of gene *B3GALT4* and *ST3GAL2* in residual dopaminergic neurons in the PD substantia nigra (Schneider, 2018), consistent with an earlier finding of decreased expression of the main brain gangliosides (GM1, GD1a, GD1b, and GT1b) in PD substantia nigra (Seyfried et al., 2018). The current study was performed to gain additional information regarding potential changes in glycoprotein and glycolipid metabolism in PD by investigating the gene expression status for select glycosyltransferases (*B3GALT4, B4GALT1, B4GALNT1, and B4GALT5*), sialyltransferases (*ST6GALNAC4, ST8SIA2, ST8SIA4, and NCAM1* (substrate for *ST8SIA2, ST8SIA4*), sialidases (*NEU1, NEU3, and NEU4*), sphingosine kinases (S1P modulators) (*SPHK1, SPHK2, and SGPL1*) (S1P lyase), and lysosomal enzymes (*GBA* (β-glucocerebroside), *GLB1* (β-galactosidase)) in the substantia nigra and putamen in patients with PD and neurologically normal controls. These genes were chosen to expand upon limited previous preliminary data that suggested potential abnormal expression levels of some glycosyltransferases, sialyltransferases, sialidases, and S1P modulators in PD brain (Schneider and Singh, 2019).

## Materials and methods

### Human brain tissue collection

Coded/anonymous substantia nigra-containing tissue blocks were obtained through the NIH NeuroBioBank and sourced from the NICHD Brain and Tissue Bank for Developmental Disorders at the University of Maryland, Baltimore, MD, the Harvard Brain Tissue Resource Center, which is supported in part by HHSN-271-2013-00030C, and from the Human Brain and Spinal Fluid Resource Center, VA, West Los Angeles Healthcare Center, 11301 Wilshire Blvd., Los Angeles, CA, which is sponsored by NINDS/NIMH, the National Multiple Sclerosis Society, and the Department of Veterans Affairs. Coded/anonymous putamen samples were obtained solely from the Human Brain and Spinal Fluid Resource Center, VA, West Los Angeles Healthcare Center. The clinical diagnosis of Parkinson’s disease was confirmed at autopsy by presence of gross depigmentation of the SN and microscopic confirmation of SN cell loss and presence of Lewy bodies in the SN and normal findings in other brain regions sampled. Frozen tissue blocks containing the SN were stored at −80°C and warmed to −20°C for dissection of samples. Dissected substantia nigra samples (containing the pars compacta region (SNc)) and dissected putamen samples (taken from dorsal putamen) were placed in sterile Eppendorf tubes and were rapidly refrozen in powdered dry ice. Standard BL2 procedures for handling human tissues were observed. Coded/anonymous non-neurological disease control tissues were obtained from the same sources mentioned above. Subject characteristics are described in Table 1 regarding substantia nigra samples and Table 2 regarding putamen samples.

**TABLE 1:** Subject and tissue characteristics: Substantia nigra.

Control	Age (yrs)	Sex	PMI (hrs)	RIN
SN1	65	M	8.8	2.7
SN2	71	M	7	2.5
SN3	83	F	8.6	3.3
SN4	65	F	8.8	4.8
SN5	70	M	18.2	2.5
SN6	77	F	6	5.7
SN7	83	F	17.6	6.1
SN8	67	F	11.8	3.1
SN9	83	M	19.5	5.9
SN10	68	M	20.3	5.9
SN11	81	F	16.3	5.9
SN12	82	M	14	6.1
**Control mean ± SEM**	74.6 ± 2.2	6 M, 6 F	13.1 ± 1.5	4.5 ± 0.5

**PD**	**Age (yrs)**	**Sex**	**PMI (hrs)**	**RIN**

SN1	76	F	8.8	4.9
SN2	79	F	9.1	4
SN3	74	M	17	4.9
SN4	79	M	27	5.5
SN5	82	F	8	6
SN6	71	M	21.5	5.7
SN7	84	M	12.7	5.5
SN8	74	M	11.4	5.7
SN9	81	F	14.8	5.6
SN10	81	F	2	2.8
SN11	67	F	22.9	5.2
SN12	78	M	9.5	3
SN13	81	M	3.5	5.5
SN14	83	M	6.7	5.4
SN15	65	M	20.3	2.8
**PD mean ± SEM**	77.0 ± 1.5	9 M, 6 F	13.0 ± 1.9	4.8 ± 0.3

**TABLE 2:** Subject and tissue characteristics: Putamen.

Control	Age (yrs)	Sex	PMI (hrs)	RIN
P1	53	M	15	6.1
P2	75	M	11.5	6.0
P3	76	M	11	6.2
P4	80	M	14	7.7
P5	61	F	26	6.2
P6	93	F	17	6.2
P7	52	M	19	5.6
P8	81	F	14.5	6.1
P9	75	F	15.4	7.3
P10	72	F	14	5.4
P11	70	M	12	5.9
P12	93	F	20.3	4.8
P13	57	M	12.6	5.0
**Control mean ± SEM**	72.2 ± 3.7	7 M, 6 F	15.6 ± 1.2	6.0 ± 0.2

**PD**	**Age (yrs)**	**Sex**	**PMI (hrs)**	**RIN**

P1	75	M	5.5	5.0
P2	72	M	5.5	4.1
P3	81	M	7	5.0
P4	75	M	5	7.1
P5	95	M	10	5.3
P6	72	F	14.7	5.7
P7	83	M	6.7	4.9
P8	67	M	9.8	6.8
P9	82	F	19	5.5
P10	70	M	22.5	5.7
P11	70	M	21.5	5.0
P12	71	M	17.5	5.2
P13	82	M	13.7	3.3
P14	81	F	12	3.0
P15	89	M	13.8	6.7
P16	73	M	30.6	5.4
P17	77	M	18.6	6.8
P18	71	M	21.5	5.3
**PD mean ± SEM**	77.0 ± 1.7	15 M, 3 F	14.2 ± 1.7	5.3 ± 0.3

### Ribonucleic acid isolation and quantitative real-time polymerase chain reaction

Ribonucleic acid (RNA) was extracted from the frozen SN and putamen samples using Zymo Direct-zol RNA miniprep Plus. During RNA isolation the DNase digestion step was performed with RNase-free DNase I (included in the kit) to eliminate genomic DNA contamination. To determine RNA quality, all the RNA samples were analyzed on an Agilent 2100 Bioanalyzer using the Agilent RNA 6000 Nano kit per the manufacturer’s instructions. The RNA integrity (RIN) numbers for the samples are reported in Tables 1, 2. cDNA was prepared using NEB Protoscript II First strand cDNA synthesis, and Real-Time PCR was performed using a Roche LightCycler 480 with Roche LightCycler 480 SYBR Green I Mastermix. Real-Time PCR was carried out using commercially sourced and validated primers from GeneGlobe Qiagen against human genes *B3GALT2, B4GALT1, B4GALT5, B4GALNT1, GLB1, GBA, NCAM1, NEU1, NEU3, NEU4, SGPL1, SPHK1, SPHK2, ST6GALNAC4, ST8SIA2*, and *ST8SIA4* (GeneGlobe IDs are provided in Supplementary Table 1). The ΔΔCt method was used to calculate mRNA expression change relative to *GAPDH* (housekeeping gene) expression.

### Statistical analyses

Raw data were subjected to outlier analysis using Grubbs test to identify and remove values that were significant outliers from the other values in each dataset. Statistical analyses were then performed using unpaired *t*-test using GraphPad Prism software (v9) with significance for gene expression change set at *P* < 0.05 (GraphPad Software, San Diego, California USA). Data were converted to fold change relative to control for graphical presentation.

## Results

Subjects and controls were well matched for age, post-mortem interval (PMI), and RNA integrity number (RIN) for both substantia nigra (Table 1) and putamen (Table 2) samples. There were no statistically significant differences between subjects and controls on any of these measures for either tissue type. Male/female ratios for substantia nigra samples were 50:50 for controls and 60:40 for subjects with PD. For putamen samples, male/female ratios were 54:46 for controls and 83:17 for subjects with PD. There were no significant sex-related differences in any of the gene expression data in either brain structure (data not shown).

**FIGURE 1:**
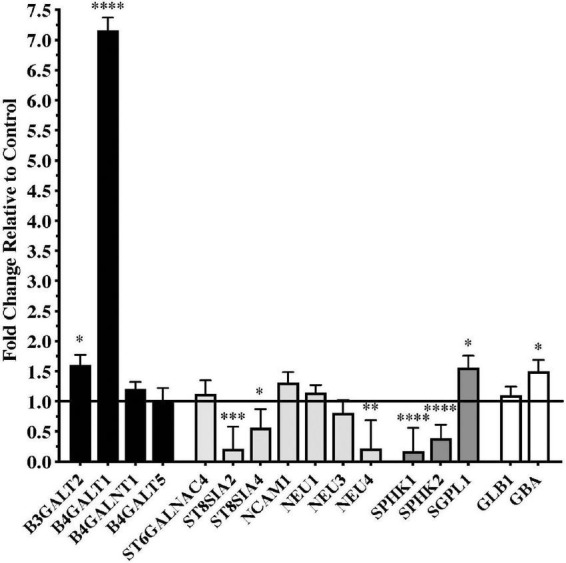
Gene expression changes in substantia nigra from patients with Parkinson’s disease (*N* = 15) relative to normal, age-matched controls (*N* = 12). Data are presented as fold change relative to control. Gene expression for glycosyltransferases *B3GALT2* and *B4GALT1* was significantly higher in patients with PD than in normal controls, while polysialyltransferase genes *ST8SIA2* and *ST8SIA4* were significantly down-regulated in the PD substantia nigra along with the gene for sialidase *NEU4*. Genes involved with sphingodine-1-phosphate (S1P) metabolism were significantly dysregulated in PD substantia nigra with expression of sphingosine kinases necessary for S1P synthesis, *SPHK1* and *SPHK2*, significantly down-regulated and gene expression for S1P lyase, *SGPL1*, involved in the degradation of S1P, was significantly up-regulated. Gene expression for glucosidase beta acid 1 (*GBA*) was also significantly up-regulated in the PD substantia nigra. *P < 0.05; **P < 0.01; ***P < 0.001; ****P < 0.0001 vs. control.

Significant differences in gene expression were found in the substantia nigra from patients with PD compared to controls. Fold changes relative to control are shown in Figure 1 for the genes assayed. Gene expression for glycosyltransferases *B3GALT2* and *B4GALT1*, the S1P modulator *SGPL1*, and the lysosomal enzyme *GBA* were significantly upregulated in PD substantia nigra. Alternatively, gene expression for polysialyltransferases *ST8SIA2* and *ST8SIA4*, sialidase *NEU4*, and sphingosine kinases *SPHK1* and *SPHK2* were significantly downregulated in PD substantia nigra.

**FIGURE 2:**
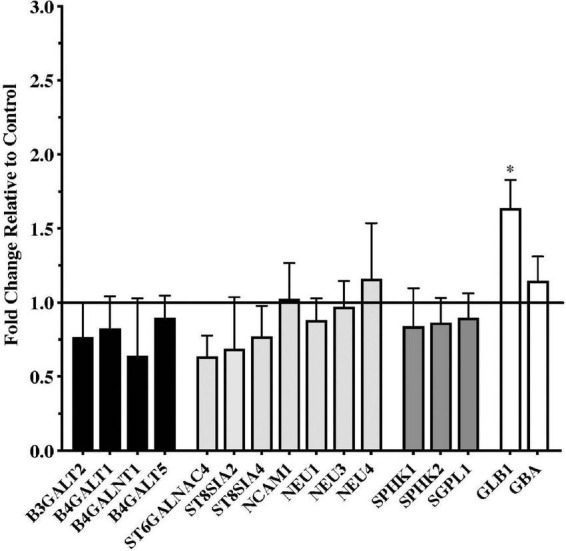
Gene expression changes in putamen from patients with Parkinson’s disease (*N* = 18) relative to normal, age-matched controls (*N* = 13). Data are presented as fold change relative to control. In contrast to the numerous changes in gene expression observed in the PD substantia nigra, expression of only one of the genes examined, the lysosomal hydrolytic enzyme β-Galactosidase (*GLB1*), was significantly altered in the PD putamen. *P < 0.05 vs. control.

Gene expression data from the putamen were overall more variable than data from the substantia nigra. Although a number of genes trended toward being downregulated in the PD samples, none of these reached statistical significance compared to the controls (Figure 2). Only one gene, the lysosomal enzyme *GLB1*, was significantly upregulated in the PD putamen (Figure 2).

## Discussion

Over the last several years, there has been an increasing appreciation for the role that gangliosides and sphingolipids in general may play in the pathogenesis and progression of neurodegenerative diseases (Maglione et al., 2010; Di Pardo and Maglione, 2018; Lansbury, 2022) and PD in particular (Chiricozzi et al., 2020; Ledeen et al., 2022; Schneider, 2022). However, less attention has been paid to the roles that possible alterations in expression of genes involved in glycosylation, sialylation, and S1P regulation may play in the development and progression of PD. The current results show significant changes in gene expression of several key molecules involved in glycosylation, sialylation, and other process relevant to sphingolipid structure and function in the PD substantia nigra.

### Glycosyltransferases

Glycosyltransferases are enzymes that catalyze the addition of polysaccharides to proteins, lipids, or nucleic acids to form glycoconjugates during glycosylation, a critical posttranslational process (Lv et al., 2017). Glycosyltransferases play important roles in the nervous system where they not only promote the development of neurons and glial cells and mediate the development of the myelin sheath (Angata et al., 2006; Lv et al., 2017) but are also critically involved in processes relevant to neurodegeneration including inflammation and microglial function, mitochondrial function, and autophagic processing (Chen et al., 2009; Videira and Castro-Caldas, 2018; Wang et al., 2020). There are various structural classes of glycosyltransferases that relate to their diverse biological functions. We previously showed that gene expression for the glycosyltransferase B3GALT4, an important enzyme in the synthesis of brain gangliosides GM1 and GD1b, was significantly reduced in residual dopaminergic neurons in the PD substantia nigra (Schneider, 2018). Currently, we show that *B3GALT2* and *B4GALT1* gene expression is significantly increased in the PD substantia nigra, compared to age-matched controls. This may be potentially significant for the expression and potentiation of PD-related pathology as protein glycosylation regulated by B4Galt1 has been suggested to be related to microglial activation and neuroinflammatory responses (Yang et al., 2020), with increased expression of B4Galt1 related to increased microglial inflammatory responses (Yang et al., 2020). Increased levels of B3GALT2 are also associated with neuroinflammation and knockdown of *B3GALT2* reduced levels of inflammatory cytokines TNFα and IL-6 (Lv et al., 2017). This is significant as microglia activation and neuroinflammatory processes have been suggested to play important roles in the pathophysiology of PD (McGeer et al., 1988; Mogi et al., 1994; Ouchi et al., 2005; Gerhard et al., 2006; Zhang et al., 2017). Further, high levels of B4GALT1 have been suggested to suppress autophagic processes (Wang et al., 2020). Impaired autophagic processing is believed to play a significant role in the accumulation of toxic α-synuclein aggregates in dopaminergic neurons in the substantia nigra and the ensuing neurodegeneration (Karabiyik et al., 2017; Miki et al., 2018; Hou et al., 2020). In contrast to the significant changes observed in the substantia nigra, there were no significant changes in glycosyltransferase gene expression in the PD putamen.

### Sialyltransferases and sialidases

Sialic acids are acidic sugars mostly found as terminal residues in glycan structures of glycoconjugates including glycoproteins and glycolipids (Rawal and Zhao, 2021). The highest levels of sialic acids are expressed in the brain where they regulate a diverse range of processes including neuronal sprouting, plasticity, myelination and myelin stability (Rawal and Zhao, 2021). Sialylation is the process mediated by sialytransferase enzymes, though which sialic acid is added to a glycoconjugate. Removal of sialic acid from sialoglycan is mediated by lysosomal, cytoplasmic, or plasma membrane bound sialidase enzymes (Schnaar et al., 2014; Rawal and Zhao, 2021). Gangliosides, sialylated glycosphingolipids that contain over 75% of the brain’s sialic acid, are the most abundant sialoglycans in the nervous system (Schnaar et al., 2014). We previously showed that gene expression for the sialyltransferase *ST3GAL2*, the enzyme responsible for the synthesis of brain gangliosides GD1a from GM1 and GT1b from GD1b, was significantly reduced in residual dopaminergic neurons in the PD substantia nigra (Schneider, 2018).

The current study also shows that there are significantly decreased levels of gene expression for two polysialyltransferases, *ST8SIA2* and *ST8SIA4*, genes that encode enzymes responsible for polysialic acid (polySia) biosynthesis. PolySia plays important roles in brain development with neural cell adhesion molecule (NCAM) as the major polySia acceptor protein (Schnaar et al., 2014). While ST8SIA2 and ST8SIA4 are important for the addition of polySia to NCAM, gene expression for *NCAM1* was not altered in the PD substantia nigra. In developing and mature brains, polySia plays roles in modulating the function of neurotrophic factors including BDNF and FGF2, NMDA and AMPA receptors, and potentially also influences dopamine and norepinephrine neurotransmission through regulating the interactions of these transmitters with their receptors (Sato et al., 2016). In addition to these functions, polySia plays a role in inhibiting innate immunity reactions, inflammation, and microglia activation (Liao et al., 2021). Thus, abnormal polysialyation could play an important role in various physiological mechanisms of relevance to the development or progression of PD. The expression of polySia is highly correlated with gene expression of *ST8SIA2* and *ST8SIA4* (Sato and Hane, 2018). ST8SIA2 has been implicated in myelin formation and ST8SIA2 deficiency leads to myelin deficits, thinning axons, and age-related white matter degeneration (Szewczyk et al., 2017). Interestingly, *St8sia2*^–/–^ mice have reduced polysialylation and display schizophrenic-like behaviors including cognitive and behavioral deficits and it was proposed that genetic variation in ST8SIA2 in humans may have the potential to confer a neurodevelopmental predisposition to schizophrenia (Krocher et al., 2015). It is not possible to know if the *ST8SIA2* gene down-regulation observed in the PD substantia nigra is a consequence of the disease or whether defects in the polytransferase gene expressions detected in our study predispose to the development of PD. While both sialyltransferases are present in the adult brain albeit at relatively low levels, ST8SIA4 appears to be the predominant polysiayltransferase in the adult brain where it has been suggested to be important for neuronal plasticity (Curto et al., 2019), with ST8SIA4 deficiency related to memory deficits in mice (Nacher et al., 2010). While the functional significance of the decreased expression of *ST8SIA2* and *ST8SIA4* genes in the PD substantia nigra are not entirely clear at this time, their dysregulation may signal a more widespread impairment in sialo-conjugate metabolism that is worthy of further study.

In addition to the decreases in mRNA expression of the polysialyltransferases discussed above, sialylation could also be influenced by the decrease expression of the sialidase gene *NEU4*. There are four main mammalian sialidases, NEU1, NEU2, NEU3, and NEU4 (Glanz et al., 2019). NEU1 is a lysosomal sialidase that participates in lysosomal exocytosis, NEU2 is primarily a cytoplasmic sialidase and plays a role in neuronal differentiation, NEU3 is a plasma membrane sialidase involved in ganglioside metabolism and regulation of transmembrane signaling, and NEU4 is located to lysosomes, mitochondria, and endoplasmic reticulum, has broad substrate specificity against sialylated glycoconjugates, and its expression has been linked to the clearance of storage materials from lysosomes, among other functions (Miyagi and Yamaguchi, 2012). In the present study, *NEU4* gene expression was significantly decreased in PD substantia nigra. In *Neu4^–/–^* mice, NEU4 has been shown to be a ganglioside metabolizing enzyme, increasing relative amounts of GD1a ganglioside while substantially decreasing GM1 levels (Seyrantepe et al., 2008). Additionally, NEU4 has been suggested to regulate neuronal function through the degradation of polySia and may also play a role on immune function in microglia (Seyrantepe et al., 2008; Takahashi et al., 2012). Together, the data presented in the current paper suggest a dysregulation of sialylation in the PD substantia nigra that could have multiple negative influences on dopaminergic neuronal function and survival. In contrast to the significant changes observed in the substantia nigra, there were no significant changes in sialylation-related gene expression in the PD putamen.

### Sphingosine-1-phosphate metabolism

Sphingosine-1-phosphate (S1P) is one the most potent signaling lipids, regulating several molecular events underlying cellular homeostasis and viability (Di Pardo and Maglione, 2018). S1P is normally synthesized by sphingosine kinase-1 and sphingosine kinase-2 (SPHK1 and SPHK2) and degraded by S1P phosphate phosphatase (SGPP) or S1P lyase (SGPL1). A balance between S1P synthesis and degradation is required for cellular homeostasis and normal cell functions (Di Pardo and Maglione, 2018). Decreased SPHK1/2 levels and increased SGPL1 levels are expected to decrease S1P levels, potentially impairing autophagic mechanisms, down-regulating pro-survival pathways, and promoting neurodegeneration (Di Pardo et al., 2019). Up-regulation of SGPL1 and reduced expression of SPHK1, with a subsequent decrease in S1P, has been associated with neurodegeneration in Alzheimer’s disease (He et al., 2010; Ceccom et al., 2014; Couttas et al., 2014) and has also been described in animal models of Huntington’s disease (HD) as well as in post-mortem brain tissues from patients with HD (Di Pardo et al., 2017). Interestingly, in HD transgenic mice, abnormally increased SGPL1 expression was observed at a very early stage of disease while SPHK1 and SPHK2 levels were not affected, suggesting that the process of dysregulation of S1P metabolism may begin very early in the disease process with alterations in expression SPHK1 and SPHK2 appearing as the disease progresses (Di Pardo et al., 2017). Importantly, pharmacological interventions aimed at modulating S1P metabolism were neuroprotective, suggesting modulation of S1P-metabolizing enzymes as potential druggable therapeutic targets for neuroprotection (Di Pardo et al., 2017). Although previous studies have demonstrated alterations in S1P metabolism using cellular and animal models of PD (Pyszko and Strosznajder, 2014; Sivasubramanian et al., 2015; Badawy et al., 2018; Motyl and Strosznajder, 2018; Zhang et al., 2018; Pepin et al., 2020) we believe the current report is the first to demonstrate this in post-mortem tissue from patients with PD. In contrast to the significant changes observed in the substantia nigra, there were no significant changes in expression of genes related to S1P metabolism in the PD putamen.

### Lysosomal enzymes

Of the lysosomal-related genes examined, only *GBA* was affected in the PD substantia nigra and only *GLB1* was affected in the PD putamen. GBA encodes for the lysosomal hydrolase β-glucocerebrosidase (GCase), that catalyzes the conversion of glucosylceramide into glucose and ceramide. Our finding of increased expression of *GBA* mRNA in the PD substantia nigra was surprising as others have reported decreased *GBA* gene expression in the substantia nigra in patients with sporadic PD (Chiasserini et al., 2015) and reduced *GBA* gene expression in brain regions with and without pathological synuclein aggregates and in early and late-stage sporadic PD (Murphy et al., 2014). The activity of GBA can be a ceramide source (Giussani et al., 2014) and ceramides play important roles in modulating membrane protein dynamics and signaling as well as modulating processes related to autophagy and mitochondrial-mediated apoptosis (Ferrazza et al., 2016). However, it is a decrease in GBA activity that is typically associated with increased ceramide levels and inhibition of autophagy and accumulation of synuclein. It is uncertain what the significance of an increase in GBA expression might be and how this may affect ceramide metabolism and accumulation as the relationship between GBA and ceramide levels is complex (Kurzawa-Akanbi et al., 2021).

β-Galactosidase (GLB1), a lysosomal hydrolytic enzyme, catalyzes the degradation of galactosylceramide to galactose and ceramide within the lysosome and *GLB1* mutation causes a deficiency in β-galactosidase-1 resulting in abnormal lysosomal accumulation of GM1 (GM1 gangliosidosis). GLB1 has not been studied extensively in PD and the significance of the increase in *GLB1* gene expression in the PD putamen is uncertain at this point.

### Study limitations

There are some potential limitations of the current study. This study utilized whole tissue extracts of substantia nigra and putamen and thus interpretation of potential gene expression changes such as the ones observed in substantia nigra homogenates from PD brain could be complicated due to loss of dopaminergic neurons and signals from other cell types (i.e., microglia). While it is not possible to know in which cell types in the substantia nigra the observed gene expression changes originated, we observed both increases and decreases in expression of specific genes and thus our data are likely not attributed solely to neuronal loss in the PD substantia nigra. There was only one gene in the PD putamen that showed a significant change in expression. This may reflect the relative contributions of the genes assessed to the pathological process that occur in the substantia nigra and not in the putamen, although the putamen gene expression data were more variable than the data derived from the substantia nigra, potentially obscuring some significant gene expression changes in the putamen. The reasons for the higher level of variability in levels of gene expression in the putamen are not entirely clear but could relate at least in part to the anatomy of the putamen and the samples made available to us for this study. The human putamen is a very large structure and although we made an effort to take all samples from the dorsal putamen, the samples came from different rostro-caudal levels of the putamen and it is possible that there are sub-regionally specific patterns of expression of the genes examined in this study in different regions of the putamen. Regional heterogeneity in expression of various neuropeptides and in dopamine innervation and gradients of dopamine transporter loss in the PD putamen are well known and this regional heterogeneity may also apply to the expression of genes currently examined. Also, a relatively small number of patient samples were examined in the current study and only a relatively small number of genes were examined. Based on the consistency of the gene expression changes observed in the substantia nigra, it is unlikely that the data are related to a potentially different gene mutation status of different patients. However, additional studies using a larger number of cases with verified gene mutation status and examining a more extensive array of genes are indicated.

### Conclusion

In summary, the current study shows significant changes in gene expression for several key molecules involved in glycosylation, sialylation, and S1P metabolism in the PD substantia nigra. Abnormal regulation of these processes has also been described in other neurodegenerative diseases including Alzheimer’s disease and Huntington’s disease, suggesting that dysregulation of processes involving glycosylation, sialylation, and sphingolipid metabolism such as those described here may transcend different brain disorders and neurodegenerative diseases.

## Data availability statement

The raw data supporting the conclusions of this article will be made available by the authors, without undue reservation.

## Ethics statement

Ethical review and approval was not required for the study on human participants in accordance with the local legislation and institutional requirements. Written informed consent for participation was not required for this study in accordance with the national legislation and the institutional requirements.

## Author contributions

JS: conceptualization and writing—original draft. GS: collection of data. JS and GS: writing—review and editing and formal analyses. Both authors contributed to the article and approved the submitted version.
