# Supplementary material for: Altered expression of glycobiology-related genes in Parkinson’s disease brain
Source: Front Mol Neurosci. 2022 Nov 24;15:1078854. doi: 10.3389/fnmol.2022.1078854 (PMC9729268; doi:10.3389/fnmol.2022.1078854)
Supplement: Supplementary file 1 [file Table_1.DOCX]

| Gene | Gene Globe ID |
| --- | --- |
| *B3GALT2* | PPH05567A-200 |
| *B4GALT1* | PPH19015A-200 |
| *B4GALT5* | PPH11065F-200 |
| *B4GALNT1* | PPH13169A-200 |
| *GLB1* | PPH21269B-200 |
| *GBA* | PPH15870B-200 |
| *NCAM1* | PPH00639F-200 |
| *NEU1* | PPH11418A-200 |
| *NEU3* | PPH19523B-200 |
| *NEU4* | PPH16523A-200 |
| *SGPL1* | PPH13925A-200 |
| *SPHK1* | PPH02491A-200 |
| *SPHK2* | PPH21192A-200 |
| *ST6GALNAC4* | PPH07231A-200 |
| *ST8SIA2* | PPH16535A-200 |
| *ST8SIA4* | PPH16711A-200 |
| *GAPDH* | PPH00150F-200 |

Supplementary Table 1. Genes and GeneGlobe Qiagen IDs for Commercially Sourced, Validated Primers.
